# Parameter Estimation of Lunar Regolith from Lunar Penetrating Radar Data

**DOI:** 10.3390/s18092907

**Published:** 2018-09-01

**Authors:** Ling Zhang, Zhaofa Zeng, Jing Li, Ling Huang, Zhijun Huo, Kun Wang, Jianmin Zhang

**Affiliations:** 1 College of Geo-exploration Science and Technology, Jilin University, Changchun 130026, China; lingzhang16@mails.jlu.edu.cn (L.Z.); inter_lijing@jlu.edu.cn (J.L.); huozj16@mails.jlu.edu.cn (Z.H.); wangkun0823@mails.jlu.edu.cn (K.W.); zjm16@mails.jlu.edu.cn (J.Z.); 2 Ministry of Land and Resources Key Laboratory of Applied Geophysics, Jilin University, Changchun 130026, China; 3 Institute of Electrics, Chinese Academy of Sciences, Beijing 100190, China; lhuang@mail.ie.ac.cn

**Keywords:** Lunar Penetrating Radar, parameter estimation, lunar exploration, regolith, data processing

## Abstract

Parameter estimation of the lunar regolith not only provides important information about the composition but is also critical to quantifying potential resources for lunar exploration and engineering for human outposts. The Lunar Penetrating Radar (LPR) onboard China’s Chang’E-3 (CE-3) provides a unique opportunity for mapping the near-surface stratigraphic structure and estimating the parameters of the regolith. In this paper, the electrical parameters and the iron-titanium content of regolith are estimated based on the two sets of LPR data. Firstly, it is theoretically verified that the relative dielectric constant can be estimated according to the difference of the reflected time of two receivers from a same target. Secondly, in order to verify the method, a parameter estimation flow is designed. Subsequently, a simple model and a complex model of regolith are carried out for the method verification. Finally, on the basis of the two sets of LPR data, the electrical parameters and the iron-titanium content of regolith are estimated. The relative dielectric constant of regolith at CE-3 landing site is 3.0537 and the content of TiO_2_ and FeO is 14.0127%. This helps us predict the reserves of resources at the CE-3 landing site and even in the entire Mare Imbrium.

## 1. Introduction

As said in Reference [1], the surface of the Moon has collided with small and large asteroidal and cometary materials for 25 billion years. As time passes, these collisions comminute the surface materials and ‘gardened’ a fine-grained layer termed “regolith.”The thickness of the regolith is between 2 and 20 m, beneath the younger maria and below the oldest surface of the lunar highland separately. Parameter estimation of the lunar regolith not only provides important information about its composition but is also critical for quantifying potential resources for lunar exploration and engineering for human outposts.

The regolith parameter could be determined by the experiments of return samples from the lunar surface. The Luna and Apollo programs have collected lunar regolith and rock samples on the lunar surface and were returned to Earth. Laboratory experiments of these samples have been conducted to reveal the nature of lunar regolith. Basu et al. [2] estimated the average chemical composition and mineral composition of lunar regolith in each sampling area, according to the lunar samples of the Apollo and Luna projects; Carrier et al. [3] and Gromov et al. [4] calculated the relationship between the mechanical properties of regolith and its density; Sen et al. [5] and Olhoeft et al. [6] measured the complex permittivity of lunar samples and fitted their relationship to density.

Some early ground-based observations and remote sensing methods also studied the nature of lunar regolith. Krotikov et al. [7] used a ground-based radio telescope to analyze the mechanical properties of lunar regolith; Tyler [8] analyzed the properties of regolith based on the Explorer electromagnetic echo; Alan et al. [9] and Pollack et al. [10] analyzed the relationship between the dielectric constant and density of large regions, such as highlands and basins, by using radar echo; Pommero et al. [11] analyzed the mineral composition, especially the iron and titanium content, with the Lunar Radar Sounder (LRS) data of the SELENE mission. These long-range detection methods can detect large areas but their accuracies are low.

The third method of parameter estimation is the in-situ detection. Lunokhod rovers and Apollo astronauts have conducted a great deal of research on the physical properties of lunar regolith but they are aimed at the mechanical properties of regolith, while the electrical properties have not been studied. On 14 December 2013, Chang’E-3 landed at 340.4875° E, 44.1189° N on the Mare Imbrium. LPR on the rover detected near-surface geological formations and the parameters of lunar regolith in the inspection area [12].

The dual-frequency Lunar Penetrating Radar aboard the Yutu Rover provides three sets of data: a set of data from the low-frequency channel (CH-1, 60MHz) to map the subsurface structure to a depth of several hundreds of meters and two sets of data (CH-2A and CH-2B) with different offsets from the high-frequency channel (CH-2, 500MHz), to detect the regolith [13].

LPR data processing and initial results are first presented by NAOC (National Astronomical Observatories, Chinese Academy of Sciences) [14]. Initial analysis of the LPR observations, especially that from CH-1, indicate that there are more than nine subsurface layers from the surface to a depth of ~360m [12]. The onboard Lunar Penetrating Radar conducted a 114m long profile, measuring a thickness of the lunar regolith layer of ∼5 m and detecting three underlying basalt units at depths of 195, 215 and 345 m. The radar measurements suggest an underestimation of the global lunar regolith thickness by other methods and reveal a vast volume from the last volcanic eruption [15]. Fa et al. [16] and Lai et al. [17] estimated the near surface structure by processing the raw CH-2 data.

Ground Penetrating Radar (GPR) is a non-destructive testing technology which is widely used in a variety of applications, such as, geophysical detection, planetary sensing, civil engineering and environmental monitoring. The main purpose is locating and imaging the hidden targets by electromagnetic detection [18]. LPR is just the application of GPR in lunar exploration. Parameter estimation of radar data is an important direction of GPR technology. A common midpoint method is the most common method of estimating the dielectric constant, which is used arange of transmitter-to-receiver offsets, assuming that the reflector is present in the ground and aligned with the midpoint between the antennas. A set of simultaneous equations involving propagation and times baseline lengths allows one to solve the dielectric constant [19]. SIMO (Single Input Multiple Output) and MIMO (Multiple Input Multiple Output) are other typical applications which are the same as this method [20,21]. The second technique involves the presence of buried point scatterers that will produce a hyperbolic radar return (the propagation time in a B-scan relative to the position of radar). The dielectric constant can be obtained by characterizing the shape of this hyperbola [22] or found by using inverse scattering algorithms with a range of prospective permittivity values [23]. The third set of techniques is trying to use a complex and often full-wave method to simulate the radar on top of the dielectric ground and coupling these with iterative optimization techniques to find the dielectric constant that matches the simulation to measurements [24,25]. In the fourth method, a more direct method can be used to design probes that penetrate the soil or extract soil samples and measure using standard laboratory permittivity [26,27,28,29,30]. The fifth approach turns to crosstalk between the transmitter and the receiver of the ground-coupled bistatic radar, with small to negligible antenna spacing. In References [31,32], the dielectric constant estimation of crosstalk frequency variation due to the presence of ground is briefly introduced. In the sixth approach, the authors estimate parameters by using the various information of the traces, such as the travel time [33], phase [34], amplitude [35], frequency spectrum [36], reflection coefficient [37], direct wave [38] and so on.

Some pre-research used LPR data to estimate the parameters of lunar regolith. Dong et al. [39] calculated the parameters of the regolith by relative reflection amplitudes; Feng et al. [40] estimate the radar velocity and the parameters of the regolith by hyperbolic matching in the CH-2B radar-gram.

In this paper, two sets of data (CH-2A and CH-2B) are used to estimate the dielectric constant of lunar regolith and the iron-titanium content. Firstly, we introduce the principle and the formula derivation of the method. Secondly, we design a flow for model verification and parameter estimation. Thirdly, a complex regolith model is carried out for the verification of this method in lunar regolith estimation. Finally, the dielectric constant and the content of TiO_2_ and FeO in lunar regolith are estimated from LPR data.

## 2. Methodology

In this paper, the parameter estimation method uses two sets of radar data with different offsets. According to the difference of the arrival time of the two sets of data from the same target, we can estimate the dielectric parameters.

This section will divide into two cases. The first case: There is no space between the radar and the ground. In the second case, there is space between the radar and the ground.

**Figure 1:**
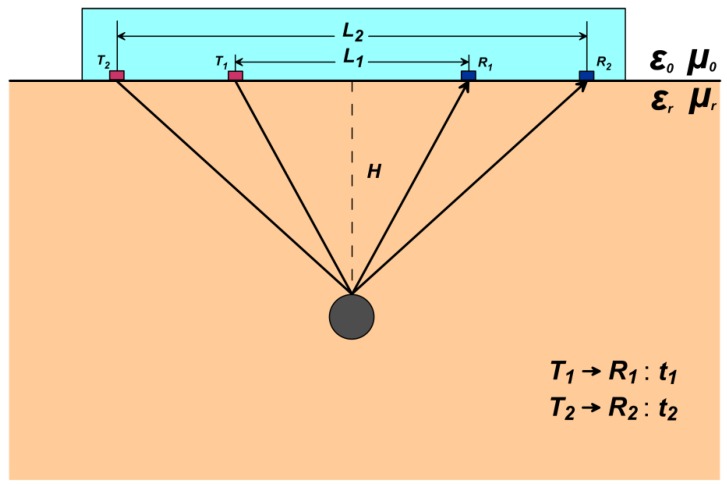
Schematic diagram of the first case (there is no space between the radar and the ground).

Figure 1 illustrates the geometric propagation paths of electromagnetic waves when the two sets of radar transmitters ($T_{1}\&T_{2}$) and receivers ($R_{1}\&R_{2}$) with different offsets ($L_{1}\&L_{2}$) are close to the ground. The dielectric constant $\varepsilon_{r}$ of the medium is estimated by the different arrival time ($T_{1}\to R_{1}$&$T_{2}\to R_{2}$) of the reflected waves from a same anomalous body.

According to the geometric relationship of Figure 1, it can be known that:

(1) $$t_{1}=\frac{\sqrt{H^{2}+{\left(\frac{L_{1}}{2}\right)}^{2}}}{v}\times 2,$$

(2) $$t_{2}=\frac{\sqrt{H^{2}+{\left(\frac{L_{2}}{2}\right)}^{2}}}{v}\times 2,$$

where $t_{1}$ is the time of the electromagnetic wave from $T_{1}$ to $R_{1}$ when the offset is $L_{1}$, $t_{2}$ is the time of the electromagnetic wave from $T_{2}$ to $R_{2}$ when the offset is $L_{2}$ and v is the velocity of the electromagnetic wave in the medium.

The relationship between the electromagnetic wave propagation velocity and the electrical parameters (dielectric constant and magnetic permeability) is known:

(3) $$v=\frac{c}{\sqrt{\varepsilon_{r}\cdot \mu_{r}}},$$

where $\varepsilon_{r}$ is the dielectric constant and $\mu_{r}$ is the magnetic permeability, c is the velocity of electromagnetic waves in a vacuum.

Obtained from Equations (1) and (3) and Equations (2) and (3):

(4) $$\frac{{t_{1}}^{2}\cdot c^{2}}{4\cdot \varepsilon_{r}\cdot \mu_{r}}=H^{2}+\frac{{L_{1}}^{2}}{4},$$

(5) $$\frac{{t_{2}}^{2}\cdot c^{2}}{4\cdot \varepsilon_{r}\cdot \mu_{r}}=H^{2}+\frac{{L_{2}}^{2}}{4},$$

and from Equations (4) and (5):

(6) $$H=\sqrt{\frac{{L_{1}}^{2}{t_{2}}^{2}-{L_{2}}^{2}{t_{1}}^{2}}{4\left({t_{1}}^{2}-{t_{2}}^{2}\right)}},$$

(7) $$\varepsilon_{r}=\frac{c^{2}\left({t_{2}}^{2}-{t_{1}}^{2}\right)}{\mu_{r}\left({L_{2}}^{2}-{L_{1}}^{2}\right)},$$

for two sets of data with different offsets, when:

(1) the transmitter and receiver are close to the ground;
(2) the offsets ($L_{1}\&L_{2}$) are known;
(3) the reflected time ($t_{1}\&t_{2}$) is known; and
(4) the medium is a non-magnetic medium ($\mu_{r}=1$),

According to Equations (6) and (7), the position of the abnormal body and the dielectric constant of the medium can be estimated.

In the actual situation, the radar transmitter and receiver are difficult to get close to the ground. Generally, there is a certain height from the ground to the radar.

**Figure 2:**
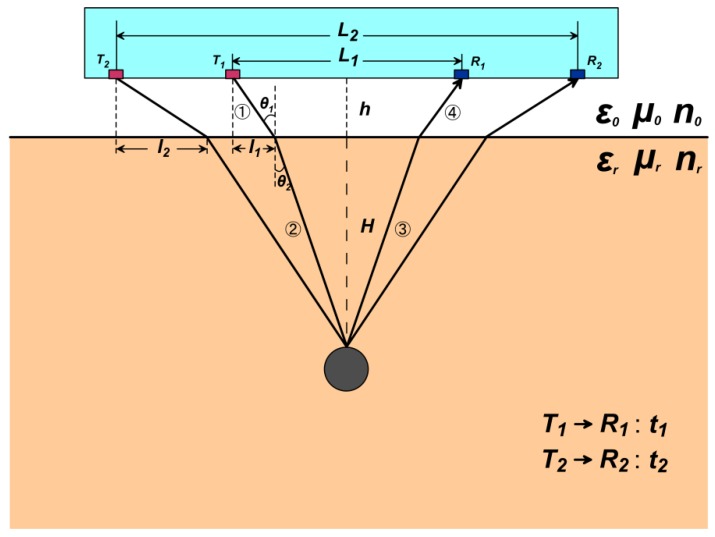
Schematic diagram of the second case (there is space between the radar and the ground).

Figure 2 illustrates the geometric propagation paths of electromagnetic waves when the two sets of radar transmitters ($T_{1}\&T_{2}$) and receivers ($R_{1}\&R_{2}$) with different offsets ($L_{1}\&L_{2}$) are at a height (h) over the ground.

Take the path from $T_{1}$ to $R_{1}$ as an example:

Snell’s Law:

(8) $$\frac{\sin\theta_{1}}{\sin\theta_{2}}=\frac{n_{r}}{n_{0}},$$

where $\theta_{1}$ is the incident angle of the electromagnetic wave, $\theta_{2}$ is the angle of refraction, $n_{0}$ and $n_{r}$ are the refractive index of the vacuum and the medium, respectively.

The velocity of electromagnetic waves in the medium:

(9) $$v=\frac{c}{n_{r}},$$

(10) $$v=\frac{c}{\sqrt{\varepsilon_{r}\cdot \mu_{r}}},$$

$n_{r}$ is the refractive index of the medium, $\varepsilon_{r}$ and $\mu_{r}$ are the relative permittivity and the relative permeability of the medium, c is the velocity of electromagnetic waves in vacuum.

According to the geometric relationship which can be seen in Figure 2:

(11) $$\sin\theta_{1}=\frac{l_{1}}{\sqrt{{l_{1}}^{2}+h^{2}}},$$

(12) $$\sin\theta_{2}=\frac{\frac{L_{1}}{2}-l_{1}}{\sqrt{{\left(\frac{L_{1}}{2}-l_{1}\right)}^{2}+H^{2}}}.$$

Bringing Equations (9)–(12) into Equation (8) and sort out:

(13) $${l_{1}}^{2}\left[{\left(\frac{L_{1}}{2}-l_{1}\right)}^{2}+H^{2}\right]=\varepsilon_{r}\mu_{r}\left(\frac{L_{1}}{2}-l_{1}\right)\left({l_{1}}^{2}+h^{2}\right).$$

According to the electromagnetic wave path and the velocity, the time from $T_{1}$ to $R_{1}$:

(14) $$t_{1}=2\left\{\frac{\sqrt{{l_{1}}^{2}+h^{2}}}{c}+\frac{\sqrt{{\left(\frac{L_{1}}{2}-l_{1}\right)}^{2}+H^{2}}}{\frac{c}{\sqrt{\varepsilon_{r}\mu_{r}}}}\right\}.$$

In the same way, we can get the relationship from $T_{2}$ to $R_{2}$:

(15) $${l_{2}}^{2}\left[{\left(\frac{L_{2}}{2}-l_{2}\right)}^{2}+H^{2}\right]=\varepsilon_{r}\mu_{r}\left(\frac{L_{2}}{2}-l_{2}\right)\left({l_{2}}^{2}+h^{2}\right),$$

(16) $$t_{2}=2\left\{\frac{\sqrt{{l_{2}}^{2}+h^{2}}}{c}+\frac{\sqrt{{\left(\frac{L_{2}}{2}-l_{2}\right)}^{2}+H^{2}}}{\frac{c}{\sqrt{\varepsilon_{r}\mu_{r}}}}\right\}.$$

In the four Equations (13)–(16), there are 11 parameters ($t_{1},l_{1},L_{1},t_{2},l_{2},L_{2},h,H,\varepsilon_{r},\mu_{r},c$). When:

(1) the offsets ($L_{1}\&L_{2}$) are known;
(2) the height (h) of the radar from the ground is known;
(3) the reflected time ($t_{1}\&t_{2}$) is known; and
(4) the medium is a non-magnetic medium ($\mu_{r}=1$).

The four remaining unknown parameters can be obtained according to the four Equations (13)–(16). Thereby, we can estimate the position of the abnormal body and the dielectric constant of the medium.

## 3. Permittivity Estimation Procedure

**Figure 3:**
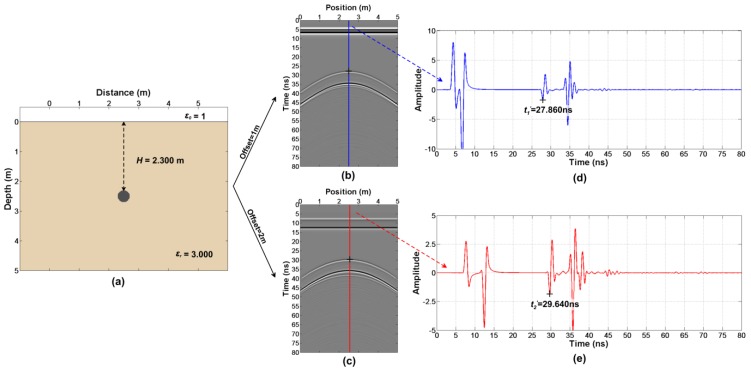
The first case (transmitter and receiver are close to the ground): (**a**) The model; (**b**) The forward result with an offset of 1 m; (**c**) Forward result with an offset of 2 m; (**d**,**e**) The single traces, as shown in the figure.

According to the following steps, the same model (Figure 3a and Figure 5a) is used to describe the above two cases.

Step 1: Simulation. Setting different parameters perform simulations of the model and we can obtain two sets of radar profiles with different offsets.

Step 2: Obtain $t_{1}$ and $t_{2}$. Read the reflection arrival time of the two sets of data from the same target. Specific operations of step 2:

Step 2.1. Firstly, read the first significant extreme point of the reflected wavelet and get ${t_{1}}^{\prime }$, ${t_{2}}^{\prime }$.

Step 2.2. Then, extract a reflected wavelet from the radar profile and read the time from the jump point to the first significant extreme point in the wavelet.

Step 2.3. Finally, the true reflected wave arrival time of the data with different offsets is obtained $t_{1}={t_{1}}^{\prime }-\Delta t$, $t_{2}={t_{2}}^{\prime }-\Delta t$.

Step 3: Parameter estimation. The dielectric constant is obtained according to the Equations (6) and (7) or Equations (13)–(16) for different situations.

The two cases are specifically described below.

Firstly, the simulation of the forward modeling (Figure 3a) have been done. FDTD (Finite-Difference Time-Domain) is applied for the simulation of the simple model. The FDTD main code was written by Irving and Knight [41] but we modified some parts of the code (the import of the model, the export of the wavefield snapshot and so on). Some key simulation parameters are presented in Table 1 and we obtain two sets of radar profiles with different offsets (Figure 3b,c).

**Figure 4:**
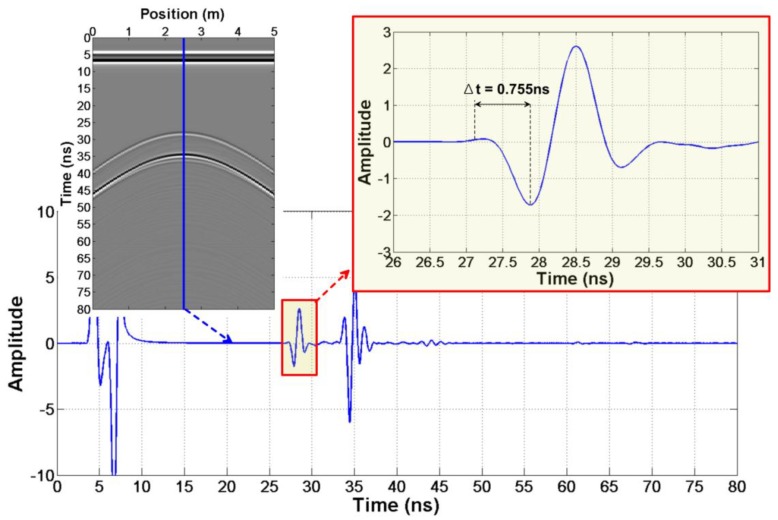
The reflection wavelet waveform of the abnormal body in the first case.

According to step 2, we firstly read out ${t_{1}}^{\prime }=27.860\;\mathrm{ns}$ and ${t_{2}}^{\prime }=29.640\;\mathrm{ns}$ in Figure 3d,e. Then, we get Δt=0.755 ns from the wavelet waveform (Figure 4). Finally, the true arrival time $t_{1}=27.105\;\mathrm{ns}$ and $t_{2}=28.885\;\mathrm{ns}$ are obtained.

**Figure 5:**
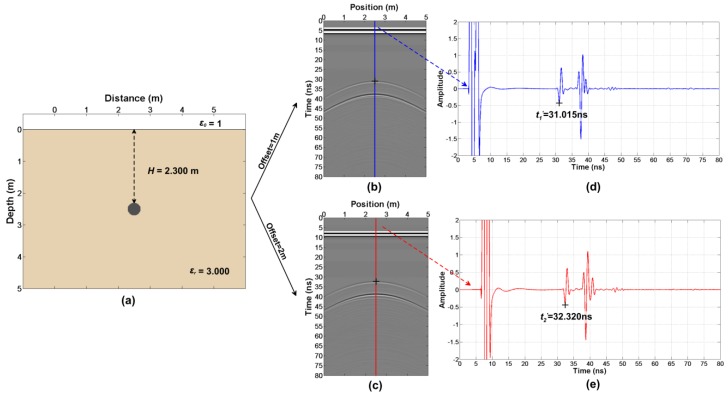
The second case (transmitter and receiver is of 0.5 m high from the ground): (**a**) The model; (**b**) The forward result with an offset of 1 m; (**c**) The forward result with an offset of 2 m and (**d**–**e**) The single traces as shown in the figure.

Consistent with the second case, the model (Figure 5a) is simulated firstly and the parameters are shown in Table 1. Two sets of radar profiles with different offsets are obtained (Figure 5b,c). What is different from the first case is that the radar transmitter and receiver is of 0.5 m above the ground.

**Figure 6:**
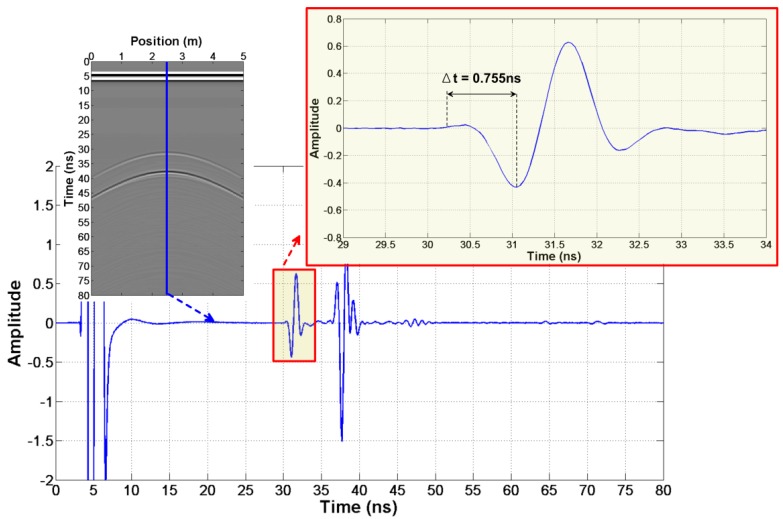
The reflection wavelet waveform of the abnormal body in the second case.

According to step 2, we firstly read out ${t_{1}}^{\prime }=31.015\;\mathrm{ns}$ and ${t_{2}}^{\prime }=32.320\;\mathrm{ns}$ in Figure 5d,e. Then, we get Δt=0.755 ns from the wavelet waveform (Figure 6). Finally, the true arrival times $t_{1}=30.260\;\mathrm{ns}$ and $t_{2}=31.565\;\mathrm{ns}$ are obtained.

Finally, following the third step, as we have already known some parameters, the quaternary equations shown below are obtained by using the Equations (13)–(16).

(17) $$\{\begin{array}{c} {l_{1}}^{2}\left[{\left(\frac{1}{2}-l_{1}\right)}^{2}+H^{2}\right]=\varepsilon_{r}\left(\frac{1}{2}-l_{1}\right)\left({l_{1}}^{2}+0.5^{2}\right)\\ 30.260\times 10^{-9}=2\left\{\frac{\sqrt{{l_{1}}^{2}+0.5^{2}}}{3\times 10^{8}}+\frac{\sqrt{{\left(\frac{1}{2}-l_{1}\right)}^{2}+H^{2}}}{\frac{3\times 10^{8}}{\sqrt{\varepsilon_{r}}}}\right\}\\ {l_{2}}^{2}\left[{\left(\frac{2}{2}-l_{2}\right)}^{2}+H^{2}\right]=\varepsilon_{r}\left(\frac{2}{2}-l_{2}\right)\left({l_{2}}^{2}+0.5^{2}\right)\\ 31.565\times 10^{-9}=2\left\{\frac{\sqrt{{l_{2}}^{2}+0.5^{2}}}{3\times 10^{8}}+\frac{\sqrt{{\left(\frac{2}{2}-l_{2}\right)}^{2}+H^{2}}}{\frac{3\times 10^{8}}{\sqrt{\varepsilon_{r}}}}\right\} \end{array}.$$

The solution, H=2.296 m, ε=2.991, which is solved by least squares, is consistent with the model. Thus, the method is feasible.

## 4. Model Experiment

In order to verify the feasibility of the method on the moon, this section conducts model tests. Two models are established: the first one is a simple model with different anomalous bodies and the second one is a complex regolith model. Verification shows that this method is feasible to estimate the electrical parameters of the regolith.

**Figure 7:**
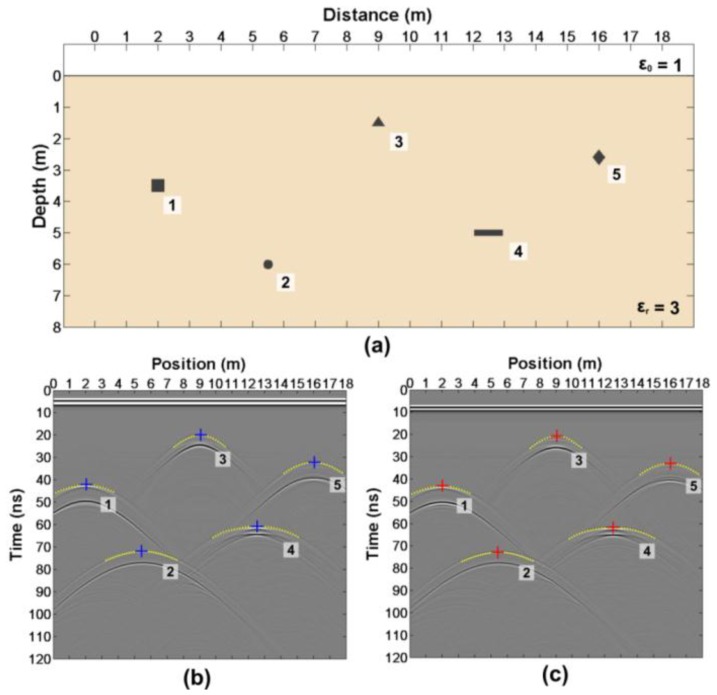
(**a**)The simple model; (**b**)The forward result with an offset of 1m; and (**c**)Forward result with an offset of 2m.

The simple model is built as shown in Figure 7a, in which there are five anomalous bodies with different shapes. The relative dielectric constant of the anomalous body is 6. Figure 7b,c shows the forward results with different offsets of 1 m and 2 m, respectively. The forward parameters are shown in Table 2.

**Figure 8:**
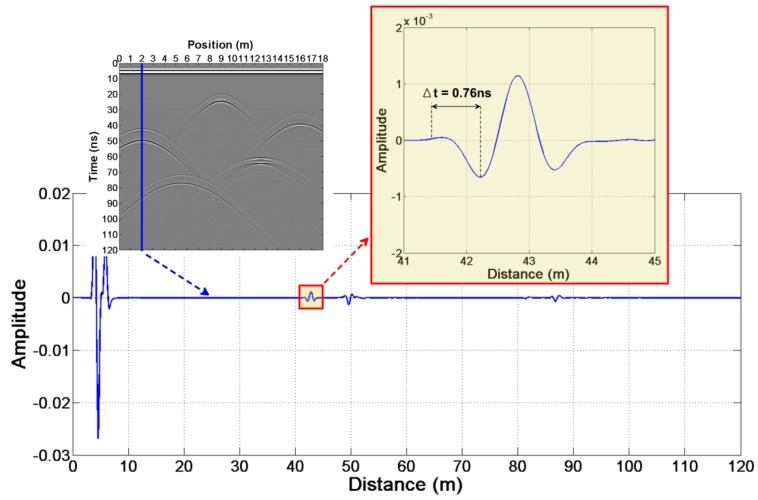
The reflection wavelet waveform of the abnormal body in the simple test model.

According to the above steps of this method, we can read the arrival time at each position (Table 3). We know Δt=0.76 ns from Figure 8. The height of the anomalous bodies and the relative dielectric constant (Table 3) can be estimated by the Equations (13)–(16).

We consider several approaches to obtain the final estimated dielectric constant:

(1) The final result is calculated by the mean of the recovered permittivity values:
  (18) $$\bar{\varepsilon }=\frac{1}{n}\sum_{i=1}^{n}\varepsilon_{i},$$
  where $\bar{\varepsilon }$ is the final estimated dielectric constant, n is the number of the recovered permittivity values and $\varepsilon_{i}$ is the *i*th recovered permittivity value.
(2) A weight based on the amplitude of the measured echoes is set:
  (19) $$\tilde{\varepsilon }=\frac{\sum_{i=1}^{n}A_{i}\cdot \varepsilon_{i}}{\sum_{i=1}^{n}A_{i}},$$
  where $\bar{\varepsilon }$ is the final estimated dielectric constant, n is the number of the recovered permittivity values and $\varepsilon_{i}$ is the *i*th recovered permittivity value. $A_{i}$ is *i*th amplitude.
(3) A weight based on the reciprocal of the estimated height of each anomalous body is set:
  (20) $$\tilde{\varepsilon }=\frac{\sum_{i=1}^{n}\frac{1}{H_{i}}\cdot \varepsilon_{i}}{\sum_{i=1}^{n}\frac{1}{H_{i}}},$$
  where $\bar{\varepsilon }$ is the final estimated dielectric constant, n is the number of the recovered permittivity values and $\varepsilon_{i}$ is the *i*th recovered permittivity value. $H_{i}$ is *i*th height.

After a comprehensive consideration, we set a weight based on the reciprocal of the estimated height of each anomalous body as the final result. Since if the standard deviation of the recovered permittivity values is quite large, calculating the mean of the recovered permittivity values is too simple and is not a reliable methodology. However, if the radar data has a low signal-to-noise ratio and a high energy attenuation, it is too difficult to obtain the real amplitude of the reflection waves. If the reference target is particularly deep, the estimation could become sensibly inaccurate, so setting a weight based on the reciprocal of the estimated height of each anomalous body as the final result could be an easy and reliable method.

The estimated dielectric constant is:

(21) $$\tilde{\varepsilon }=\frac{\sum_{i=1}^{n}\frac{1}{H_{i}}\cdot \varepsilon_{i}}{\sum_{i=1}^{n}\frac{1}{H_{i}}}=2.9792,$$

which is consistent with the model.

**Figure 9:**
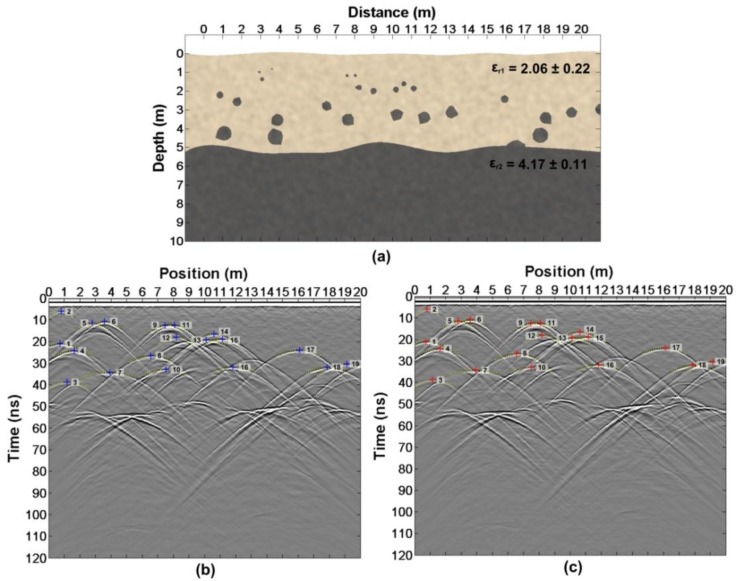
(**a**) The complex model; (**b**) the forward result with an offset of 1 m;and (**c**) the forward result with an offset of 2 m.

**Figure 10:**
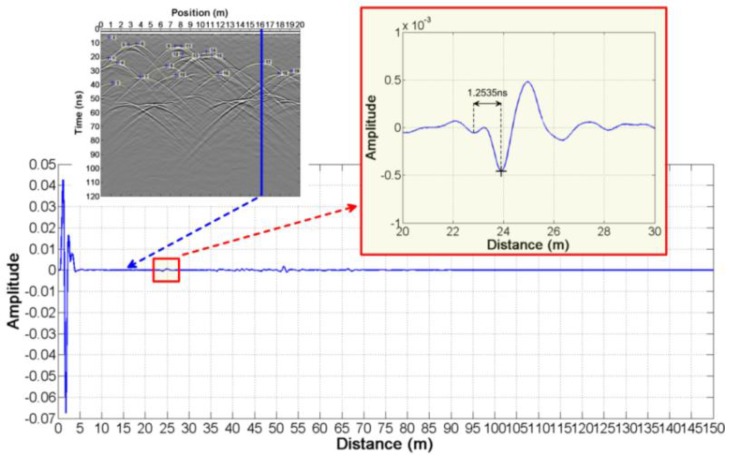
The reflection wavelet waveform of the abnormal body in the complex test model.

In order to verify whether this method is suitable for LPR data, we build a complex model (Figure 9a). This model considers many factors: random medium, undulating interface and anomalous body. The modeling method is referenced in References [42,43,44]. According to the actual acquisition parameters of LPR [13], the simulated parameters are shown in Table 2. Two sets of forward results with different offsets are obtained in Figure 9b,c. According to the above method, reading from the two sets of radar data, ${t_{1}}^{\prime }$ and ${t_{2}}^{\prime }$ are shown in Table 4. Δt=1.2535 ns is from Figure 10. The height and the relative dielectric constant (Table 4) are estimated according to the Equations (13)–(16).

It can also be found that for this kind of complex data the variance of the result is relatively large (the standard deviation is quite large) due to the non-uniformity of the medium, so, as mentioned above, we set a weight based on the reciprocal of the estimated height of each anomalous body as the final result. The estimated final dielectric constant is:

(22) $$\tilde{\varepsilon }=\frac{\sum_{i=1}^{n}\frac{1}{H_{i}}\cdot \varepsilon_{i}}{\sum_{i=1}^{n}\frac{1}{H_{i}}}=2.1050.$$

This is consistent with the forward complex model ($\varepsilon_{r1}=2.06\pm 0.22$).

## 5. Result

**Figure 11:**
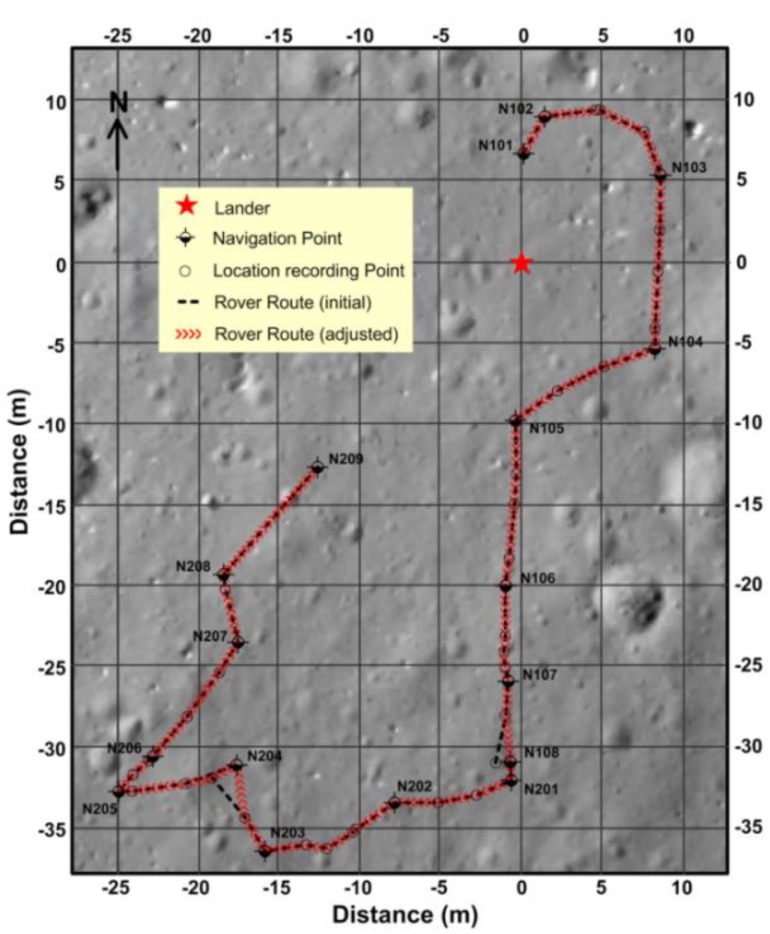
Yutu’s path on the Moon. The context image was taken by the descent camera on the CE-3 lander. The red star shows the landing site. The inset lines show the initial path (black dotted line) read directly from the Lunar Penetrating Radar (LPR) data and the adjusted path (red line).

The Yutu rover released by CE-3 was the first soft landing on the Moon since the Soviet Union’s Luna 24 mission in 1976. To be specific, the Yutu rover explored the surface and subsurface of the landing site in the northern part of Mare Imbrium using its four main instruments: The Panoramic Camera, Lunar Penetrating Radar (LPR), Visible–Near Infrared Spectrometer (VNIS) and Active Particle-Induced X-ray Spectrometer (APXS). Its track extends to 114.8 m (Figure 11) near a young crater. In this part, the data processing results of the LPR are reported.

**Figure 12:**
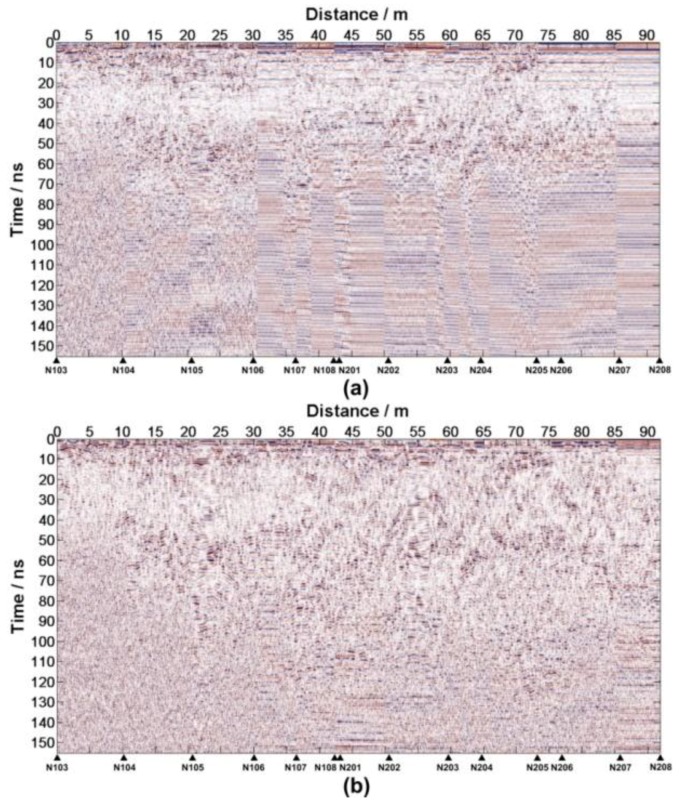
(**a**) The LPR CH-2A image and (**b**) The LPR CH-2B data image. N103-N209 denote the positions where the LPR was rebooted.

Aiming at the near-surface stratigraphic structure of the regolith, the CH-2 data is selected. TheCH-2 has two receiver antennas (CH-2A and CH-2B).The LPR data processing pipeline is designed according to the acquisition parameters, the actual situation and the data quality (Table A1 in Appendix A). Two radar images with high resolution (Figure 12) are accessible after data editing and processing. The IDs for the data from Lunar Penetrating Radar are listed in Table A2 in the Supporting Materials. 

**Figure 13:**
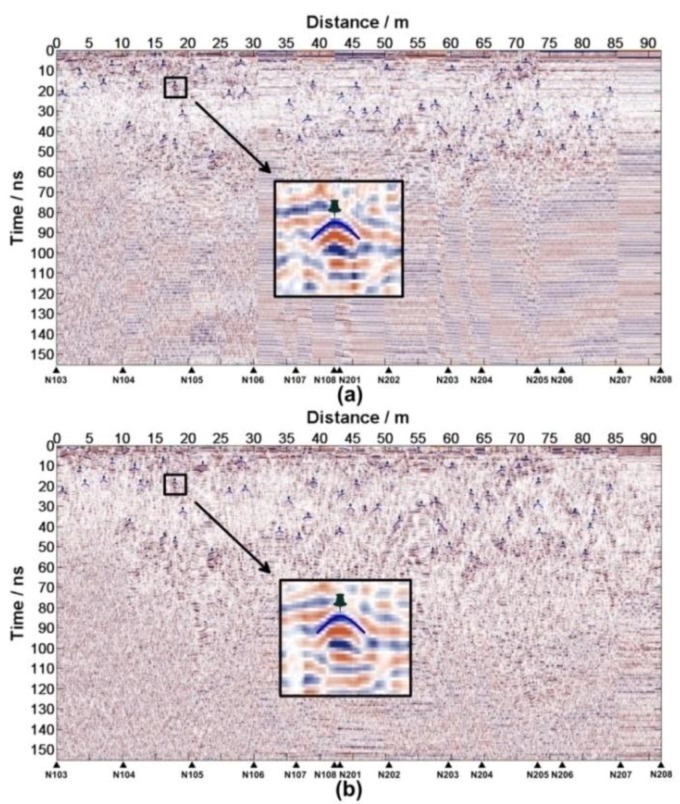
(**a**) Reflected waves caused by basalt blocksshown in CH-2A data and (**b**) Reflected waves caused by basalt blocksshown in CH-2B data.

**Figure 14:**
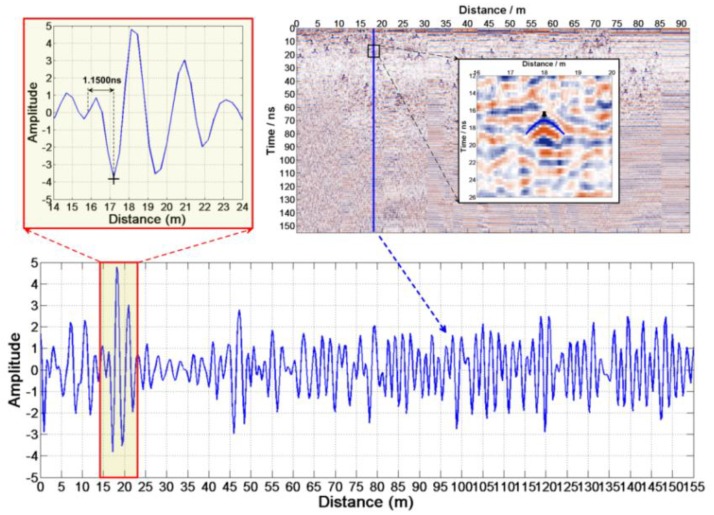
The reflection wavelet waveform of an abnormal body in LPR data.

At first, the reflected waves caused by the same basalt block should be found in CH-2A and CH-2B data (Figure 13). Reading the ${t_{1}}^{\prime }$ and ${t_{2}}^{\prime }$ of each position follows according to Figure 14, Δt=1.1500 ns. Equations (13)–(16) estimate the height and the relative dielectric constant of each anomaly body, which are shown in Table A3.

**Figure 15:**
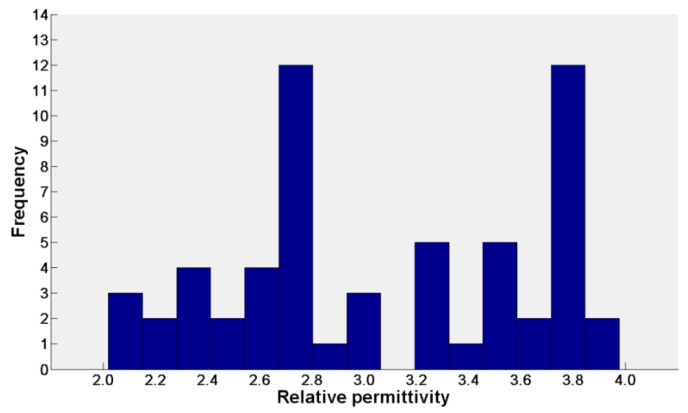
The histogram of the recovered permittivity.

In order to support the conclusion in this paper, deep statistical analysis of the results should be conducted. A histogram of the recovered permittivity is shown in Figure 15.

If we only focus on the analysis of the recovered permittivity data (ε in Table A3), the mean value of the estimated dielectric constant is μ=3.0537, the standard deviation is σ=0.5923. However, as with the previous analysis, we believe that if the weight based on the reciprocal of the estimated height of each anomalous body is applied, the estimated dielectric constant is much more accurate:

(23) $$\mu =\tilde{\varepsilon }=\frac{\sum_{i=1}^{n}\frac{1}{H_{i}}\cdot \varepsilon_{i}}{\sum_{i=1}^{n}\frac{1}{H_{i}}}=3.0109,$$

and the standard deviation:

(24) $$\sigma =\sqrt{\frac{1}{n}\sum_{i=1}^{n}{\left(\varepsilon_{i}-\mu \right)}^{2}}=0.5887.$$

We believe that the recovered permittivity obeys the normal distribution, the estimated final dielectric constant is ε=3.0109±1.1538 (confidence level at 95%).

According to the above result, a reasonable dielectric constant result has been given but it is found from Figure 15 that the recovered dielectric constant distribution is not a standard normal distribution and the standard deviation is also relatively large. We consider it is due to the heterogeneity of the regolith medium. The recovered dielectric constant is not only related to the location but also related to the depth. An analysis between permittivity and the depth (or location) of the target should be performed to improve data post-processing.

**Figure 16:**
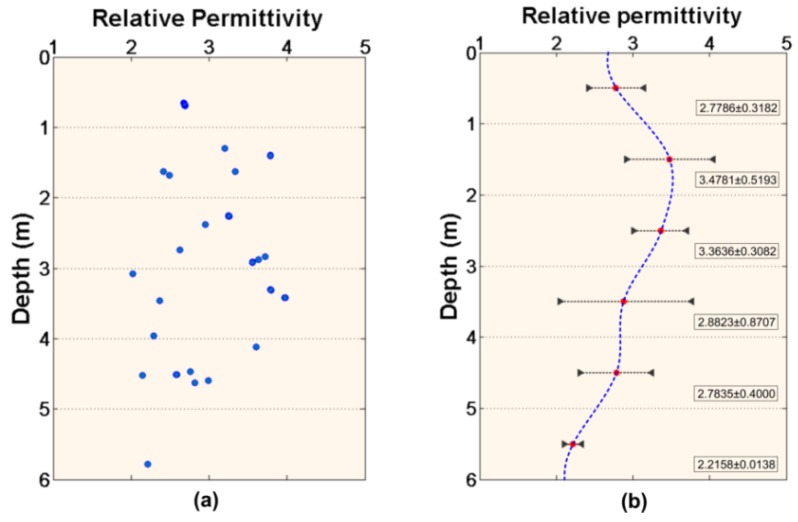
Analysis between permittivity and the depth. (**a**) Scatterplot of permittivity and depth. (**b**) The average estimated dielectric constant of regolith at each meter. The error bars show the standard deviation.

**Figure 17:**
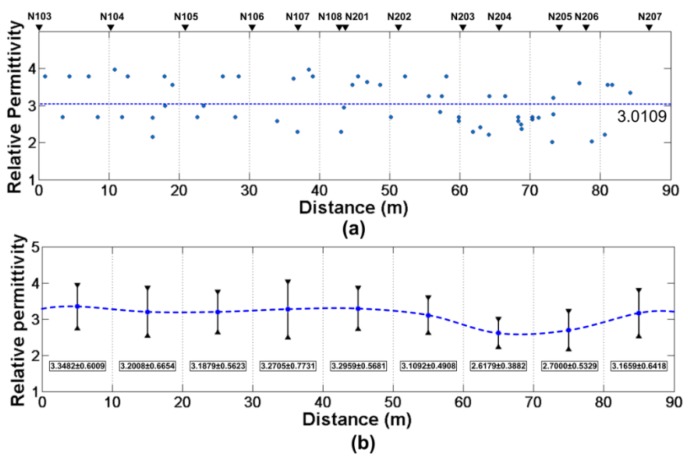
Analysis between permittivity and the location. (**a**) Scatterplot of permittivity and distance. (**b**) The average estimated dielectric constant of regolith at each 10 m. The error bars show the standard deviation.

Figure 16a is scatterplot of permittivity and depth and at each meter, we calculate the mean values and the standard deviations (Figure 16b). The figure shows us a relationship between permittivity and depth. There is a maximum value at ~2m, which is due to the stratigraphic structure at the CE-3 landing site. At the same way, the analysis between permittivity and the location of LPR has been performed in Figure 17. The permittivity does not change drastically with location.

The lab test results from lunar regolith samples on Apollo and Luna era show that the dielectric constant of lunar regolith is related to the density of lunar regolith [1]. The density is yielded by the dielectric constant as follows:

(25) $$\rho =\log_{1.919}\varepsilon_{r}.$$

The loss tangent (tan *δ*) can be easily yielded by the density as follows:

(26) $$\tan\delta =10^{\left(0.440\rho -2.943\right)},$$

and:

(27) $$\omega (TiO_{2})+\omega (FeO)=\mathrm{lg}(\tan\delta )-0.312\rho +3.260.$$

**Figure 18:**
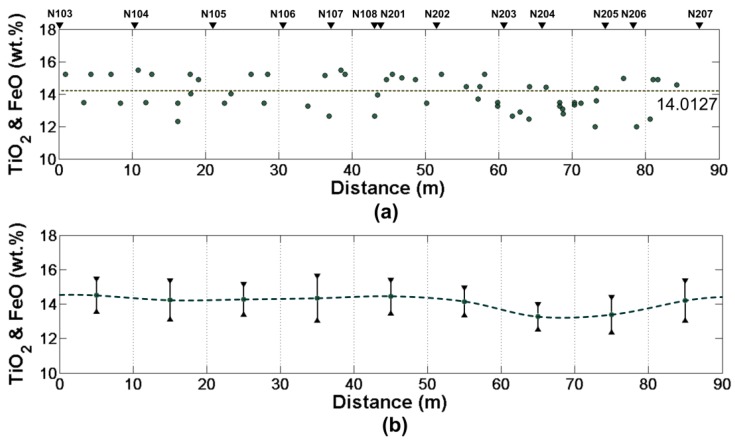
Analysis of the content of $TiO_{2}$ and FeO and the location. (**a**) Scatterplot of the content of $TiO_{2}$ and FeO and distance. (**b**) The average content of $TiO_{2}$ and FeO every 10 m. The error bars show the standard deviation.

On the basis of the obtained relative dielectric constant, On the basis of the obtained relative dielectric constant, the content of TiO_2_ and FeO on CE-3 landing site were estimated (the results are shown in Figure 18).

After the analysis, we consider the estimated relative permittivity of lunar regolith at 3.0109 and the content of TiO_2_ and FeO is 14.0127%, which is consistent with other methods [3,39,40].

## 6. Discussion

The method for permittivity estimation in this paper has the advantages compared with the method mentioned in the introduction:

(1) It is easier to understand and operate. This method uses two sets of data with different offsets to estimate the permittivity. The main work is reading the arrival times of the same anomalous body. It is easier to operate than the methods in the introduction, since the various methods need to extract complex information, such as amplitude, spectrum, phase and so on.
(2) The data quality requirements are lower, even if the radar data contains various types of noise, so as to affect the data waveform, amplitude and frequency, it will not affect the applicability of this method.
(3) No prior information and other data are needed. This method does not require any a priori information, such as incident wave information and does not require other data, such as TDR (Time-Domain Reflectometry) data and so on.
(4) It is more suitable for the LPR data collected by CE-3 mission. The original intention of this method is only for the parameter estimation of LPR data. The before methods depend on the low signal-to-noise ratio of the data and need to have all kinds of accurate information, while LPR data is collected on the moon, various types of noise affect the signal-to-noise ratio of the data and deep processing, including gain, filtering, background removal and so forth, has to be done which affected the nature of the data. The method in this paper only needs the information of the arrival time to perform parameter inversion.

However, it is undeniable that this method also has disadvantages compared to other methods. Two sets of data with different offsets are required. It is necessary to extract an accurate wavelet. Only the dielectric constant can be estimated and the conductivity is ignored. This method is only applied in the case of containing various types of scatterers.

Since this method is mainly aimed at the inversion of the lunar soil parameters, it is still a good predictor for the parameters of the lunar regolith.

There are also many factors which will affect the accuracy of the result when using this method for the parameter estimation:

(1) Sampling rate. Horizontal and vertical sampling rate will determine the resolution of the radar profile, which will affect the accuracy.
(2) Offset. The distances of transmitters and receivers will also affect the result. In general, the larger the offsets are, the more accurate the result will be.
(3) The height of the radar.
(4) Wavelet extraction. Selecting a good standard wavelet waveform will affect our reading, which will further affect the result.
(5) Noise. The signal-to-noise ratio will also affect the accuracy of the data and affect the result of parameter estimation.
(6) The heterogeneity of the medium. If the medium is strong non-uniformity, the accuracy of the result will be affected.
(7) Personal error. When reading various types of data, personal error cannot be ignored.

An important direction of radar development is single-input multiple-output (SIMO) and multiple-input multiple-output (MIMO), therefore the data with different offsets can be obtained. It is also an important boost for this method which can estimate the position of the abnormal body and the dielectric constant of the medium. In order to avoid the damage to the regolith collector, the Chang’E-5 (CE-5) mission of China will install a MIMO radar on the lander which will help to detect the nature of the regolith and the position of the underlying abnormal rocks on the landing site. The above method will also be able to apply to the CE-5 radar data for the analysis of the stratigraphic structure on the landing site.

## 7. Conclusions

According to the geometric propagation law of electromagnetic waves, the propagation paths of two different electromagnetic waves are used to deduce the position of the anomalous body and the electrical parameters of the medium. The method is proved to feasible in theory. When focus on two sets of radar data whose offset are different, the dielectric constant of the medium is inferred by the two different reflection time of a same anomalous body. The process of the parameter estimation method is feasible. A simple model and a complex model of lunar regolith is simulated for the verification of the method. The estimated parameters are consistent with the models.

The LPR data collected by CE-3 mission whose CH-2 data are with different offsets give us the chance to estimate the dielectric constant of regolith on the landing site. The results of the Apollo program show that the dielectric constant is intrinsically linked to the density and the iron-titanium content of regolith. Combined with the estimated dielectric constant from LPR data, the density and the TiO_2_ and FeO content are obtained.

Compared with the parameters estimated method of by Apollo’s samples, the application of Lunar Penetrating Radar for parameter estimation has a larger range and lower cost. Compared with the remote sensing estimation, the LPR can be more accurate. The parameters of regolith estimated by LPR data help us predict the reserves of resources at the CE-3 landing site and even the entire Mare Imbrium.

## Figures and Tables

**Table 1:** Simulation Parameters.

	First Case		Second Case	
**Height**	0 m		0.5 m	
**Offset**	1 m	2 m	1m	2m
**Center frequency**	500 MHz	500 MHz	500 MHz	500 MHz
**Waveform**	Ricker	Ricker	Ricker	Ricker
**Absorbing boundary**	C-PML		C-PML	
**Discrete grid**	0.005 m × 0.005 m		0.005 m × 0.005 m	
**Time step**	0.040434 ns		0.040434 ns	
**Time window**	80 ns		80 ns	
**Random access memory**	8.00 GB		8.00 GB	
**Central Processing Unit**	Intel(R) Core(TM) i5-4590 CPU @3.30GHz			
**Time**	4.5371 h	4.6237 h	4.5825 h	4.6333 h

**Table 2:** Simulation parametersof the experiment models.

	Simple Model		Complex Model	
**Height**	0.5 m		0.3 m	
**Offset**	1 m	2 m	0.16m	0.32m
**Center frequency**	500 MHz	500 MHz	500 MHz	500 MHz
**Waveform**	Ricker	Ricker	Ricker	Ricker
**Absorbing boundary**	C-PML		C-PML	
**Discret** **e grid**	0.005 m × 0.005 m		0.005 m × 0.005 m	
**Time step**	0.040434 ns		0.040434 ns	
**Time window**	120 ns		120 ns	
**Random access memory**	8.00 GB		8.00 GB	
**Central Processing Unit**	Intel(R) Core(TM) i5-4590 CPU @3.30GHz			
**Time**	14.2794 h	14.3112 h	20.5625 h	20.6101 h

**Table 3:** Estimated parameters of the simple experiment model.

Number	${t_{1}}^{\prime }$	${t_{2}}^{\prime }$	H	$\varepsilon_{r}$
1	42.21	43.22	3.2917	2.9581
2	71.69	72.33	5.8370	2.9957
3	19.73	21.51	1.3041	2.9608
4	60.85	61.58	4.9433	2.9373
5	31.98	33.27	2.3579	3.0407

**Table 4:** Estimated parameters of the complex test model.

Number	${t_{1}}^{\prime }$	${t_{2}}^{\prime }$	H	$\varepsilon_{r}$
1	21.0257	21.0662	1.8446	2.0855
2	6.0247	6.1055	1.1901	2.1815
3	38.5337	38.5741	2.2442	2.6161
4	24.2604	24.3009	2.0058	2.4644
5	11.6046	11.6854	1.318	2.5777
6	10.8768	10.9172	1.5026	1.4297
7	34.2072	34.1668	3.0825	1.7297
8	26.4439	26.6056	2.8795	2.1557
9	12.4537	12.4941	1.057	1.8755
10	32.7516	32.8325	3.9485	2.0367
11	12.4133	12.4941	1.3554	2.6194
12	17.9527	18.0336	2.0002	1.8514
13	19.2466	19.3275	2.2046	1.8754
14	16.5375	16.6184	1.535	2.718
15	18.9231	18.9636	1.7318	1.8389
16	31.9025	31.9834	3.9499	2.1793
17	23.8965	24.0987	3.298	1.4519
18	31.8621	31.9429	3.7867	1.637
19	30.366	30.4064	3.6163	1.6606

## Appendix A.

**Table A1:** Explanation of data processing.

	Processing	Explanation
i	Data reading	Data is divided into 9 sections. According to their storage format (*.psd, the standard storage format in aviation and spaceflight field), the data and location information are read one by one.
ii	Data stitching	Data is divided into 9 sections, which should be spliced into a group.
iii	Traces amending	Not only the differ of section but also the error of collection start time can make the Image longitudinal displacement. Based on the phase of a strong reflection event, we adjust the traces.
iv	Traces selecting	The rover patrolled with non-uniform motion, since the rover might stop at some points on the way to collect other scientific data such as APXS data, VNIS data and so on. However, LPR never stop acquisition, which resulted in repeated acquisition of multiple traces at same location. Stack and average the repeated traces. The first and second sections with low SNR (Signal to Noise Ratio) should be deleted.
v	Time lag adjustment	The arrival time of the radar echo was delayed by 28.203 ns corresponding to the start time for recording data.
vi	Useless data deleting	The first 150 ns data with great research value need to be aimed in, since the rest of data has low SNR.
vii	Band-pass filter	Eliminate noise by band-pass filter.
viii	Background removal	In order to highlight the abnormal information, subtract the average trace.
ix	Automatic gain control	AGC (Automatic Gain Control) has been to observe the bottom information.
x	Re-positioning	Add location information to the image.

**Table A2:** IDs of the LPR data (Data are hosted at http://moon.bao.ac.cn).

IDs
CE3_BMYK_LPR-2A_SCI_N_20091231160000_20131215171000_0001_A.2B
CE3_BMYK_LPR-2A_SCI_N_20131215171001_20131220141300_0002_A.2B
CE3_BMYK_LPR-2A_SCI_N_20131220141301_20131220181800_0003_A.2B
CE3_BMYK_LPR-2A_SCI_N_20131220181801_20131221124500_0004_A.2B
CE3_BMYK_LPR-2A_SCI_N_20131221124501_20131223174500_0005_A.2B
CE3_BMYK_LPR-2A_SCI_N_20131223174501_20131226000000_0006_A.2B
CE3_BMYK_LPR-2A_SCI_N_20131226000001_20140112193800_0007_A.2B
CE3_BMYK_LPR-2A_SCI_N_20140112193801_20140114213300_0008_A.2B
CE3_BMYK_LPR-2A_SCI_N_20140114213301_20140124000000_0009_A.2B
CE3_BMYK_LPR-2B_SCI_N_20091231160000_20131215171000_0001_A.2B
CE3_BMYK_LPR-2B_SCI_N_20131215171001_20131220141300_0002_A.2B
CE3_BMYK_LPR-2B_SCI_N_20131220141301_20131220181800_0003_A.2B
CE3_BMYK_LPR-2B_SCI_N_20131220181801_20131221124500_0004_A.2B
CE3_BMYK_LPR-2B_SCI_N_20131221124501_20131223174500_0005_A.2B
CE3_BMYK_LPR-2B_SCI_N_20131223174501_20131226000000_0006_A.2B
CE3_BMYK_LPR-2B_SCI_N_20131226000001_20140112193800_0007_A.2B
CE3_BMYK_LPR-2B_SCI_N_20140112193801_20140114213300_0008_A.2B
CE3_BMYK_LPR-2B_SCI_N_20140114213301_20140124000000_0009_A.2B

**Table A3:** Estimated parameters of LPR data.

Number	Position	${t_{1}}^{\prime }$	${t_{2}}^{\prime }$	H	ε
1	0.92	21.5625	22.1875	1.4063	3.7888
2	3.42	11.5625	12.8125	0.6976	2.6942
3	4.36	17.5000	18.7500	1.4002	3.7867
4	7.16	15.9375	17.1875	1.3978	3.7858
5	8.42	7.1875	8.4375	0.6743	2.6857
6	10.82	37.5000	38.7500	3.4219	3.9751
7	11.86	9.6875	10.9375	0.6848	2.6906
8	12.68	17.8125	18.7500	1.3986	3.7861
9	16.20	4.6875	5.6250	0.6539	2.6761
10	16.22	44.0625	45.6250	4.5178	2.1483
11	17.96	17.1875	18.4375	1.4009	3.7869
12	18.00	45.9375	47.1875	4.5962	2.9888
13	19.04	31.5625	32.8125	2.9142	3.5546
14	22.58	9.0625	10.0000	0.6785	2.6876
15	23.48	52.1875	53.1250	4.5977	2.9904
16	26.20	21.5625	22.8125	1.4062	3.7888
17	28.00	6.5625	7.8125	0.6709	2.6842
18	28.48	20.6250	21.8750	1.4048	3.7883
19	33.96	40.9375	42.1875	4.5069	2.5813
20	36.26	25.0000	25.9375	2.8346	3.7183
21	36.88	41.5625	42.8125	3.9599	2.2931
22	38.46	34.3750	35.6250	3.4198	3.9734
23	39.00	16.8750	18.1250	1.399	3.7863
24	43.10	41.5625	42.1875	3.9594	2.2924
25	43.50	23.1250	24.0625	2.3842	2.9531
26	44.70	29.6875	30.9375	2.9129	3.5537
27	45.50	17.8125	18.7500	1.3983	3.786
28	46.80	27.8125	29.0625	2.8759	3.6363
29	48.62	29.6875	30.9375	2.9129	3.5537
30	50.14	8.7500	9.6875	0.6768	2.6868
31	52.20	35.6250	36.5625	3.307	3.7909
32	55.52	25.0000	26.2500	2.2627	3.2549
33	57.20	51.2500	52.1875	4.6225	2.8207
34	57.40	24.0625	25.3125	2.2615	3.254
35	58.06	37.1875	38.1250	3.3085	3.7921
36	59.80	48.7500	50.0000	4.5091	2.5841
37	59.84	9.6875	10.9375	0.6842	2.6903
38	61.86	37.1875	38.1250	3.9582	2.2909
39	62.90	20.9375	21.8125	1.6263	2.4147
40	64.06	54.3750	55.6250	5.7778	2.2159
41	64.16	29.6875	30.6250	2.2678	3.2589
42	66.44	23.4375	24.3750	2.2574	3.2507
43	68.26	9.3750	10.6250	0.6829	2.6897
44	68.30	46.2500	46.8750	4.5068	2.5811
45	68.70	18.4375	19.3750	1.683	2.4902
46	68.80	36.2500	37.1875	3.4646	2.3693
47	70.30	31.2500	32.1875	2.7392	2.628
48	70.32	9.3750	10.6250	0.6829	2.6897
49	71.20	5.9375	6.8750	0.6604	2.6792
50	73.08	28.1250	29.0625	3.0756	2.021
51	73.26	16.8750	17.8125	1.2966	3.2006
52	73.30	41.5625	42.5000	4.4647	2.7566
53	76.96	48.4375	49.6875	4.1149	3.6006
54	78.80	31.8750	32.8125	3.0782	2.0245
55	80.60	51.8750	53.4375	5.7777	2.2157
56	81.00	35.6250	36.5625	2.9159	3.5557
57	81.66	32.8125	34.0625	2.915	3.5552
58	84.26	19.6875	20.6250	1.6266	3.3369
